# Electrospun Nanofibers for Dental Regeneration Therapy: Advancements and Perspectives

**DOI:** 10.34172/apb.46505

**Published:** 2026-05-19

**Authors:** Pritha Dalapati, Vanshita, Inderbir Singh

**Affiliations:** Chitkara College of Pharmacy, Chitkara University, Punjab, India

**Keywords:** Electrospun, Nanofibers, Dental regeneration, Bone tissue engineering

## Abstract

**Purpose:**

This review discusses recent progress in electrospun nanofibers for dental regenerative treatments. It covers their fabrication techniques, characterization methods, and diverse applications such as pulp-dentine regeneration, periodontal therapy, bone tissue engineering, dental implants, wound healing, and drug delivery. Additionally, the review summarizes patents, clinical studies, and future outlooks to evaluate the potential of electrospun nanofibrous systems in dentistry.

**Methods:**

A narrative review was conducted, examining scientific papers, patents, and clinical trial data related to electrospinning techniques and dental regeneration. The review evaluated studies on polymer choice, electrospinning settings, scaffold characterization, and dental uses. Key findings from experimental, preclinical, and clinical studies were integrated to analyze current advancements and challenges.

**Results:**

Electrospun nanofibers exhibit a biomimetic structure, high porosity, and customizable physical properties, making them ideal for dental engineering. They enhance cell adhesion, growth, and differentiation, as well as enable controlled drug release. Their uses include antimicrobial periodontal membranes, bioactive pulp-dentin scaffolds, osteoconductive bone regeneration matrices, implant coatings that improve osseointegration, and wound dressings that accelerate healing. Several patented methods and early clinical trials show encouraging results, especially in periodontal drug delivery and bone regeneration.

**Conclusion:**

Electrospun nanofibers are a highly adaptable and potent choice for dental regeneration due to their resemblance to the natural extracellular matrix and their capacity to embed bioactive agents. Although notable advancements have been made, issues such as mechanical stability, large-scale production, and long-term biocompatibility remain challenging. Ongoing innovation in smart fibers and hybrid scaffolds is crucial for effective clinical application.

## Introduction

 The dental tissues, enamel, dentin, cementum, and periodontal ligament do not have very strong self-healing abilities, so their restoration to full functionality and structural integrity is a significant challenge for regenerative dentistry.^1^ Traditional methods focused on the repair rather than true regeneration, and these are fillings, crowns, implants, and periodontal surgeries used to be an area. All these traditional ways of restoring dental tissues focused on repairing damaged parts and did not fully restore them to their original architecture and biomechanical properties. With more recent advances in tissue engineering, it has been possible to biologically regenerate dental tissues in different ways through the use of biomaterials (scaffolds) to instruct the behavior of cells where they are directed to be placed, enhance the ability of the dental tissues to become integrated, and assist with the development and transference of therapeutic agents.^2^ In the field of tissue engineering, electrospun nanofibers have garnered a lot of attention because of their ease of manufacturing and ability to be customized in terms of pore size, scaffold structure, and fiber alignment. These reasons have led to the recent use of polymeric nanofibers in dentistry, where their flexibility and nanostructure have facilitated highly favorable cell homing behaviors, raising hopes for improved tooth regeneration.^3^ Because of their physicochemical and biological characteristics, such as osteoconductive and bioactivity, which are very similar to those of dental tissues, bioceramics have demonstrated excellent biocompatibility with dental tissues and teeth.^4^ Electrospun nanofibers can also mimic the nanoscale structure of the extracellular matrix (ECM), which provides a biomimetic microenvironment that enhances cell adhesion, proliferation, migration, and differentiation. The ability of electrospun nanofibers to regulate the physical and chemical characteristics based on the design of the scaffold includes a variety of characteristics such as fiber diameter, porosity, fiber orientation, and functionalization. Custom-designed scaffolds for specific dental tissues (pulp, dentin, periodontal ligament, and bone) are available through the many combinations of these characteristics.^5^

 Important examples of natural polymers used to make nanofibers include collagen, cellulose, silk fibroin, keratin, gelatin, and polysaccharides like chitosan and alginate. Collagen is an extracellular protein that occurs naturally and provides structural integrity to numerous connective tissues. Its fibrillary structure, which ranges in diameter from 50 to 500 nm, is essential for cell adhesion, recognition, proliferation, and differentiation. It was shown that the developed collagen scaffold enhanced cell adhesion and reduced cell migration as the fiber diameter increased using electrospun collagen nanofibers.^6^ The primary problem with natural polymers, however, is that when cells recognize them, they can easily cause an immune response. Thus, synthetic polymers like as polycaprolactone (PCL), poly (lactic acid) (PLA), poly (lactic-co-glycolic acid) (PLGA), polyurethane (PU), poly(L-lactide) (PLLA), and others have been developed as alternatives to scaffold inclusion.^7^ Due to their biodegradability and biocompatibility, these synthetic polymers can be used to produce matrices with fiber diameters in the nanoscale range.^8^ In addition, bioactive, antibacterial, and drug-loaded nanofibrous scaffolds have been developed over the past few years to support tissue regeneration and address some of the most difficult issues associated with tissue regeneration, including inflammation and infection and vascularity. Growth factors, stem cells, nanoparticles, synthetic polymers, and natural polymers are incorporated into the nanofibers, further enhancing their therapeutic utility. Newer and more refined methods of producing scaffolds, such as multilayered scaffolds, coaxial electrospinning, and 3D-aligned fiber constructs, improve the mechanical strength and functional efficiency of the scaffold.^9^

 This review provides a comprehensive overview of nanofibers, with a focus on the electrospinning technique. This article summarizes various dental regeneration applications and discusses various nanofibers that are developed with the interactions of polymers and drugs. Furthermore, it discusses the patents and clinical trials and identifies the future perspective.

## Fabrication Method of Nanofibers

 Nanofibers are ultrafine structures having a diameter of less than one micrometre. Due to their unique properties, they are widely used in biomedical engineering, drug delivery, wound healing etc. Fabrication method plays a crucial role in defining properties of nanofibers for their specific applications.^10^ Nanofiber fabrication may be roughly classified into two methods: top-down and bottom-up. While in bottom-up approach nanofibers are formed by putting together materials to create larger nanomaterials and in top-down approach bulk materials are broken into extremely small nanosized particles which are then used to create nanofibers.^11^ Bicomponent fixer splitting, melt blowing, physical drawing, flash-spinning, gel (dry wet) spinning, air jet spinning, phase separation, self-assembling, solvent dispersion, centrifugal spinning, hydrothermal, and electrospinning are just a few of the many nanofiber production processes that have been developed. Depending on the intended fibre quality and application, each manufacturing technique has pros and cons of its own. Several variables, including porosity, chemical composition, fibre diameter, and mechanical strength, affect the procedure selection.^12^

 Electrospinning is the most widely used technology out of all of them due to its benefits, including affordable instruments, straightforward manufacturing, little material needed, and potential for industrial production. Electrospinning techniques use electrostatic forces to produce fibers from a polymer solution. High voltage and electric charge are used in the process to deform a polymer solution into conical droplets, which are then expelled from the nozzle tip as tiny fibers with a diameter of 10–10,000 nanometers.^13^

 The electrospinning system is a quite straightforward and approachable technique. A high-voltage power source to charge the solution of polymer, a syringe pump to pass the solution via a capillary, which is connected to a syringe filled with the solution of polymer, a spinneret (hypodermic needle), and a collector to capture nanofibers are the 4 main parts (Figure 1).^14^

**Figure 1:**
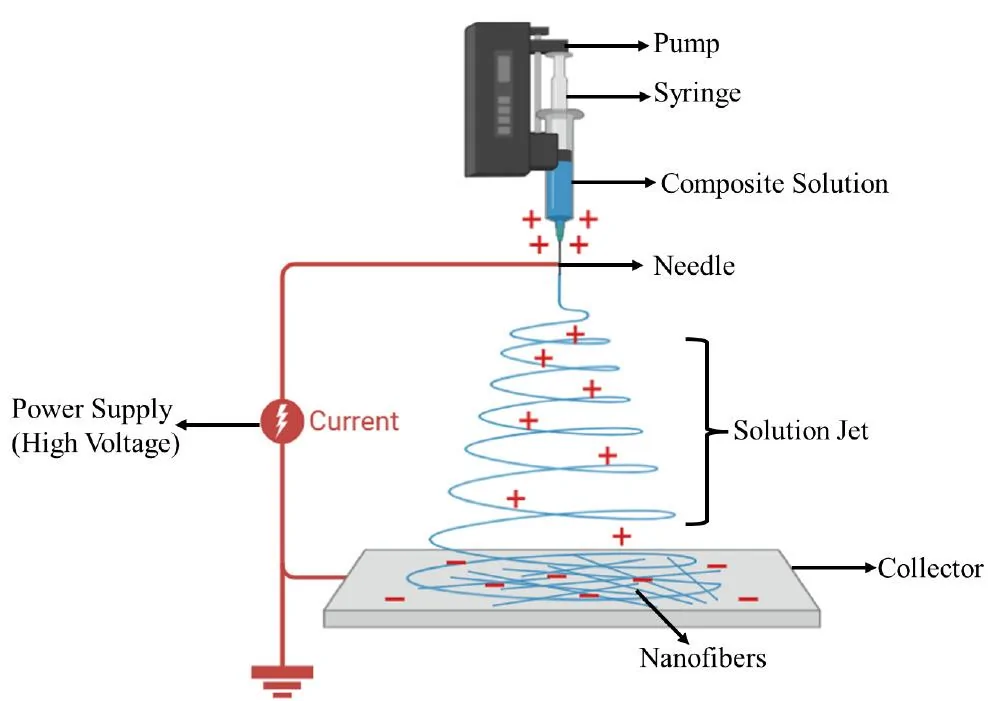
Development of nanofibers using electrospinning technique

 There are four steps in the electrospinning process. First, the liquid droplet is charged and forms a Taylor cone; next, the charged jet extends along a straight line; next, the jet’s diameter decreases near the electric field and whipping instability develops; and finally, the jet solidifies due to solvent evaporation and accumulation on the collector.^15^

 In electrospinning, due to surface tension, the liquid is forced out of the spinneret and forms into a pendant droplet. When the polymer solution is electrified, the electric charges are developed and accumulated on the surface, and later the charges with the same sign on the surface start to repel each other, and when this electrostatic repulsion prevails over the surface tension, a Taylor cone is formed from the pendant droplet, and through this cone charged jet is expelled.^13^ Now, due to bending instability, the jet extends in a straight line before undergoing whipping motion. This results in a decrease in the diameter of the jet, due to which the jet solidifies quickly and gets deposited on the collector. When the solution has high viscosity, the jet goes to the ground collector as fiber jets rather than breaking up into droplets, as seen in the case of the low viscosity solutions.^16^

 There are two possible configurations for the electrospinning apparatus: vertical and horizontal. The primary difference between uphill and downhill electrospinning setups in vertical is the orientation of the fibers and the number of beads. When compared to the downward technique’s unpredictable orientation, the fiber collection from the upward method will have a uniform arrangement. Also, the formation of beads in fibers in the case of the upward method will be less compared to the downward method.^17^

 Factors affecting Electrospinning process-

 Morphology of the electrospun nanofibers depend upon certain parameters (Figure 2), these are divided into three broad categories: solution parameters, process parameters, and ambient parameters are discussed in Table 1.

**Figure 2:**
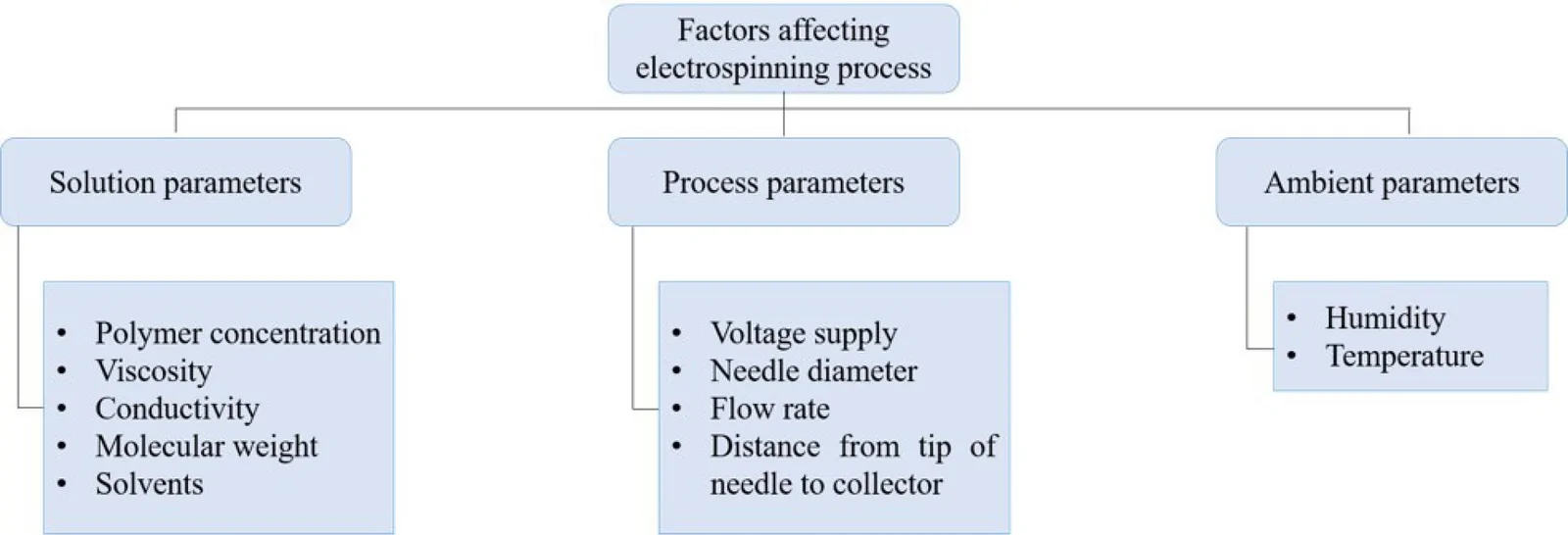
Various factors viz. critical solution, process, and ambient factors that affect the electrospinning process

**Table 1:** Polymer Solution, Processing, and Ambient Parameters Influencing Electrospun Nanofiber Fabrication

**Polymer Solution Parameters**		**Ref**
Polymer Concentration	Low concentration of polymer solution leads to beads formation in the fibers while increasing the conc. leads to pure straight fibers	^18^
Surface tension	When the solution's surface tension is reduced, beads free fibers are developed	^19^
Viscosity	Less viscous polymer solution forms beads while at high viscosity extrusion of solution becomes difficult	^20^
Conductivity	Highly conductive polymer solution is quite unstable which leads to broad distribution of it and this small diameter fibers are obtained at the collector	^21^
Molecular weight	Increase in molecular weight decreases the beads formation within fibers.	^22^
Solvents	Suitable solvents for fine fibers are those which have moderate boiling point and can dissolve polymer properly	^23^
**Processing Parameter**		
Applied Potential difference	Higher potential difference value leads to decreased diameter of the fibers	^24^
Rate of flow	A high flow rate causes the diameter and pore size to rise as well as the production of beaded fibers	^25^
Diameter of needle tip	Nanofibers with a narrow normal distribution and a tiny diameter are produced when the needle's diameter is decreased	^26^
Collectors Morphology	While aligned fibers are acquired by utilizing a target/collector with a consistent rotation speed or by using specifically designed collectors, such as spinning drums, a random mat of fibers will be created from the basic collector plate	^27^
Distance from needle tip to collector	The fibers diameter dropped to its minimal value as the distance rose until an optimal value was attained, but after that, it increased as the distance and applied voltage increased	^28^
**Ambient Parameters**		
Humidity	Increasing humidity results in porous and thicker nanofibers	^29^
Temperature	Higher temperature results in thinner fibers	^30^

## Characterization of Nanofibers

 To comprehend electrospun nanofibers shape, mechanical characteristics, chemical makeup, and functional performance, characterization is crucial. The primary methods utilized for their characterisation are listed below.

**Mechanical properties: **A texture analyzer is used to measure mechanical qualities such as elongation and tensile strength. The fibers are trimmed to 25 mm in length and 5 mm in breadth. A thickness tester was used to measure the fiber thickness. Two grips were used to secure three samples for each fiber ratio, which were then stretched along the fiber axis at rate of 0.01 m/min. The following formula was used to get the tensile strength and elongation of the fibres.^31^


$$\begin{array}{l} Tensile\;strength\;of\;fibers\;\left(MPa\right)=\frac{Force\;applied\left(N\right)}{Cross\;sectional\;area\;of\;fibers\;\left(mm^{2}\right)}\\ Elongation\;of\;fibers\;\left(\%\right)=\frac{\Delta L\times 100}{L} \end{array}$$

**SEM:** By examining a sample’s surface, scanning electron microscopy (SEM) can provide details about its morphology. This is often accomplished by directing a beam of electron at the sample, which causes excitation of atoms present on the surface and secondary electrons are released. After identifying these, an image can be created. The ability of SEM to qualitatively evaluate cell development on surface layers gives it an advantage over physical techniques. SEM’s maximum achievable resolution is around 1 nm. SEM pictures help to evaluate apparent density and compare it to the bulk density of the electrospun polymer, SEM is also used to evaluate pore diameter, fiber diameter, fiber alignment and porosity. After being sliced into 0.5 × 0.5 cm square strips, the nanofibers are adhered to the sample table. To make the surface conductive and prevent charge, samples are frequently nanospun with gold, platinum, or other conductive elements. SEM was used to view the nanofibers’ fiber structure at a 6.0 kV accelerating voltage.^32^

**Wettability: **The rate of wound healing is impacted by the hydrophilicity of nanofibers, which also affects cell adhesion and growth behaviors. Additionally, during the healing phase, it is important to apply and adhere wound dressing membranes to the moist wound bed. A drop shape instrument was used to evaluate the wettability, which was indicated by polarity, contact angle and surface free energy, in order to ascertain the hydrophilicity of the produced fibers. The Wu’s harmonic mean equation is used to compute the polarity, contact angle and surface free energy after two liquids—used as nonpolar and polar solvents—are dropped on each fiber mat throughout the procedure.^33^

**Antimicrobial capacity: **The disk diffusion method is used to assess the antibacterial efficacy of nanofibers against both gram-positive and gram-negative bacteria. The nanofibers are chopped into 1cmdiameter circular sections. The plates are filled with the LB agar medium. The bacterial suspension is then applied to the LB agar plates that have hardened. The plates were covered with the prepared samples, three of each type. The plates are then cultivated for 24hr at 37 °C in an incubator. The diameter of the inhibitory zone is then measured three times, and the average value is determined.^34^

**Absorbency: **Wound mats are ideal dressing materials because they should keep the surface of the wound wet and absorb exudates. The following formula was used to investigate and compute the wound dressing’s degree of swelling (DS):

 DS = (W_w_−W_d_) × 100 /W_d_

 Where,

 W_w_: wet dressing weight after immersion in the medium for the specified durations (1, 5, 10, and 15 minutes)

 W_d_: dry dressing weight

 The media utilized in this piece replicated the conditions of a chronic wound. Two sieves are used to separate the samples. By drying in hot air oven at 50 °C and using filter paper, the extra fluid is eliminated. Using an experimental design technique, the maximum absorbency values at time point following medium immersion were chosen and assessed.^31^

## Dental Regeneration Applications

 In order to preserve and restore the biological vitality of teeth and gums, dental regeneration is linked to tissue engineering and other methods that replace or repair lost or damaged dental tissues, including enamel, dentin, and even entire teeth. As previously observed, electrospun nanofibers have a number of uses in dental regeneration. A few of these are listed below:

## Pulp-Dentine Regeneration

 Dental trauma is a prevalent issue that primarily affects children aged 8-12 worldwide. Their teeth are more vulnerable to dental damage due to their open apices, larger pulp chambers, and weaker dentin walls. Therefore, tooth damage may allow bacteria to enter the root canal and contribute to the degeneration and necrosis of dental pulp tissue, resulting in pulp necrosis and vascular-nerve bundle rupture.^34^

 In regenerative endodontics, new nanotechnological methods, particularly nanofibers, have gained popularity because they share characteristics with the lost tissue; they can create efficient drug-delivery systems to combat microorganisms in the root canal system. To restore this tissue,nanofibers made from various polymers such as PCL, PLA, and PGA are used.^35^ While enabling new tissue to develop and be supported by these scaffolds, the aim is to eliminate the majority of germs from the root canal system.

 Patients with endodontic disease will have an alternative to root canal therapy or tooth extraction, as endodontic treatment can maintain the pulp’s function to some degree and permit further root growth.^36^ For such severe diseases, there are now two treatment options: vital pulp therapy if the pulp is still vital, or root canal therapy if the pulp is permanently inflamed. Vital pulp treatment aims to use scarred tissue to form a dentinal bridge, maintaining pulp vitality and function after reversible pulpal damage. Electrospun scaffolds that can stimulate odontoblast regeneration have been investigated to enhance and expedite the outcomes of pulp therapy.^37^ Electrospun scaffolds of hydroxyapatite (HA) and polyvinyl alcohol were created by Kim et al. and may have dentin regeneration potential.^38^ Additionally, electrospun PCL meshes have shown a great deal of promise for promoting odontogenic differentiation and development when tested *in vitro* using human pulpal cells, as evidenced by increased turnover of collagen I and other proteins.

 Numerous electrospun scaffolds containing various medications were created by Bottino et al. They created polydiaxonone (PDS) electrospun scaffolds with antibiotics (ciprofloxacin and metronidazole) added to the solution. It was shown that these scaffolds could deliver antibiotics more effectively and required lower doses to fight dangerous bacteria such as *Porphyromonas gingivalis* and *Enterococcus faecalis* than drugs delivered via pastes. In addition to promoting the development and adhesion of cells derived from human pulp, the three-dimensional porous scaffolds also deliver drugs to the pulp cavity and the root canal.^39^ Electrospinning has made it possible to create bioactive three-dimensional scaffolds made of PDS and halloysite aluminosilicate clay that can repair the pulp dentin complex by delivering materials like growth hormones and antibacterial drugs. The favourable effects of apatite-mineralized electrospun polycaprolactone nanofiber scaffolds were promising for dental tissue engineering, as evidenced by evaluations of cell adhesion, proliferation, and odontoblast differentiation. Additionally, adding growth agents like nerve growth factor can significantly promote pulp regeneration.^40^

## Periodontal Regeneration

 The periodontium is composed of the gingiva, cementum, alveolar bone, and periodontal ligament (PDL). All regions of the periodontium are affected by periodontitis, a multifactorial, non-communicable inflammatory disease that causes irreparable damage and is defined by the gradual, chronic deterioration of the tooth-supporting machinery.^41^ Around 15% of persons globally have periodontal disease, which is caused by plaque biofilm accumulation at the gingival–tooth contact. After spreading into the subgingival niche, this biofilm destroys periodontal tissue, which eventually leads to tooth loss.^42^ Regenerative techniques, such as bone transplants and membranes for guided tissue regeneration (GTR), are necessary to replace the lost tissues in advanced stages of the disease.^43^ Recently, electrospinning has been used to construct fibrous and porous biodegradable scaffolds, such GTR membranes.

 In order to avoid periodontal inflammation and arrest tissue degradation, early regimens mostly concentrated on mechanical scaling and root planing, with adjuvant treatment with antibiotics or antiseptics employed seldom. Because uncontrolled drug release techniques would not meet physiological demands and naked growth factors or other molecules may be easily disseminated or rapidly deactivated, periodontal regenerative therapy requires more advanced drug delivery technologies. This may lead to undesirable consequences, such as cancer and dysbiosis.^44^ Because of the amazing developments in nanotechnology, a variety of organic or inorganic nanoplatforms have been considered as viable options for nano-based drug delivery systems (nano-DDSs), which can be essential in overcoming the present challenges in tissue regeneration.^45^

 Mechanically removing the majority of the disease-causing biofilm above and below the gum line and encouraging an environment with fewer bacteria for tissue repair are the two main objectives of periodontal therapy. Additionally, the prognosis and restoration of periodontal health are improved by the use of antibacterial agents in conjunction with mechanical treatment.^46^ Given their dual roles as antimicrobial chemical release mechanisms and a guide for the development of new tissues, nanofibers have emerged as a potential therapy option for periodontal disease.^47^

 Because of their special qualities, including high porosity, surface area, and interconnectivity, nanofibrous scaffolds have been extensively investigated in tissue engineering (TE) procedures. Electrospinning makes it possible to efficiently create nanofibrous scaffolds that can mimic the size and form of native extracellular matrix (ECM) proteins in order to support cell adhesion, proliferation, and differentiation.^48^

 Electrospun nanofiber mats, which may be utilized as drug delivery systems to reduce the depth of periodontal pockets and enhance the oral health of patients with chronic periodontal disease, have been successfully treated with antibacterial drugs such as metronidazole and chlorhexidine.^49^

 Since electrospun fibrous scaffolds are ideal for the creation of periodontal GTR barrier membranes and biomimetic scaffolds, electrospinning has been utilized in a variety of TE methods, including periodontal TE. A key characteristic of a GTR membrane is its high porosity and tiny hole size, which can stop fibroblast migration across the nanofibrous scaffolds. As an alternative to bone grafts, electrospun nanofibrous scaffolds can be incorporated into a TE construct that promotes tissue regeneration by offering a more accurate representation of the natural extracellular matrix.^50^ Nanofibrous scaffolds can satisfy the requirements of an ideal GTR membrane, including being biodegradable, biocompatible, osteoinductive, and possessing good mechanical properties, by utilizing optimum electrospinning conditions and a combination of carefully chosen synthetic and natural polymers.^51^

 Apart from their similarity to natural extracellular matrix, the scaffolds can be functionalized with growth factors and ceramics to improve their biological effects by including biomolecules or applying surface coatings. To mimic the native inorganic bone component and boost the scaffold’s bioactivity and osteoconductivity, hydroxyapatite, a significant component of natural bone, can be added to the fibers. These nanofibrous scaffolds may be able to attract host stem and progenitor cells and encourage their proliferation and differentiation into fibroblasts, osteoblasts, and cementoblasts, potentially regenerating all of the periodontal tissues, due to their increased bioactivity and close resemblance to the native extracellular matrix.^52^

 For example, the PCL electrospun fibrous membrane doped with 0.5% (w/v) zinc oxide (ZnO) exhibits remarkable osteoconductivity and antibacterial properties in rat periodontal defect tests. The functionally graded membrane filled with platelet-derived growth factor (PDGF) may effectively stimulate alveolar ridge regeneration, prevent wound dehiscence, and speed up the regeneration process.^53^

## Bone-Tissue Regeneration

 With an inner cancellous bone and an exterior cortical bone, bones are a hierarchical structure. Its essential functions protection, support, mobility, and hematopoiesis are determined by its distinct and intricate structure. Once bone is damaged by trauma, infection, or tumor removal, its continuity and integrity may be lost. The process of bone healing is very complex and affected by various biological factors. Treating bone repair and regeneration in clinical settings often presents major challenges, especially for maxillofacial bone defects.^54^

 In contrast to traditional tissue transplantation, which includes autografts and allografts, bone tissue engineering procedures eliminate the problems of donor inadequacy, supply constraints, and immunological rejection. It involves scaffolds, stem cells, and growth factors making it a promising approach for bone repair and regeneration. Scaffolds are used in conjunction with cells and biological supplements in tissue engineering techniques. One of the most important problems in bone tissue engineering is the development of a bio-artificial bone implant, a scaffold that mimics the extracellular matrix (ECM) and has osteoconductive properties, that restores damaged or diseased bones.^55^

 Electrospun nanofibers are considered ideal scaffolds for bone tissue engineering because of their varied structures and functions.^4,56^ Their diameters closely match those of the natural extracellular matrix (ECM), providing a favorable environment for cell adhesion, growth, movement, and differentiation. Additionally, these nanofibers have a large surface area and high porosity, making them effective for loading different bioactive substances and enabling controlled drug release.^16^

 To encourage bone formation, natural or synthetic scaffolds containing osteogenic stem cells are inserted into bone defect regions. Human body cells are extracted and isolated, a certain number of cells are cultured *in vitro*, the cells are packed into scaffolds, and the scaffolds are then implanted into the bone deformities. The goal of combining scaffolds, growth factors, and stem cells is to promote bone defect healing.^57^

 The scaffolds’ performance is critical to BTE’s success. To restore bone integrity, the scaffolds should degrade at the appropriate time to allow for bone growth, support pressure, and encourage osseointegration.^58^ Several electrospun nanofibrous scaffolds for bone regeneration made of natural and synthetic polymers, with or without mineral deposition.^59^ Gelatin-PCL, silk-fibroin-PCL, PLA, gelatin-apatite-poly (lactide-co-caprolactone), mesoporous silica-shelled PCL, mesoporous bioactive glass-incorporated PCL-gelatin, and PCL nanofibrous scaffolds composed of magnetic nanoparticles are a few examples.^60^ Jain et al.^61^ developed curcumin-loaded PCL electrospun nanofibers (CU1 and CU5) to study how drug release affects osteogenesis and compared these with a drug-free PCL scaffold (CU0). Both fiber mats released about 18% of the drug by day 3. By day 6, CU1 and CU5 showed drug releases of 42% and 50%, respectively. In vitro tests with MC3T3-E1 mouse pre-osteoblasts indicated that ALP activity followed the order of CU1 > CU0 > CU5. The optimal curcumin concentration and sustained release in CU1 enhanced osteogenic expression compared to CU5, which had a higher drug loading content. Additionally, several polymeric nanofibers have been discovered and used as cellular substrates for bone; however, they lack bioactivity and other biofunctionalities that could hasten bone tissue regeneration. This was accomplished by introducing artificial mineralization after manufacturing or by adding additives (such as growth factors and bioactive nanoparticles) to scaffolds during electrospinning. This accelerated natural mineralization, vascularization, and induced osteogenesis.^62^ These nanofibrous scaffolds would be employed as a carrier for bone-associated growth factors because of their 3D networked pores, which make it easier to regulate drug release.^63^

## Dental Implants

 Dental implants, also known as osseointegrated appliances, are surgical devices that maintain permanent or removable prosthodontic and orthodontic equipment in direct contact with the bone.^64^ Dental implants have been made from a variety of materials, including titanium and its alloys. The usage of zirconia and reinforced polymers, like polyetheretherketone, as dental implants have increased recently.^65^ Because metal implants are physiologically inert by nature, they have drawbacks, such as the inability to form a strong enough chemical bond with the surrounding bone, particularly in the early stages of implantation. The most studied methods involve developing porous implant surfaces and coatings.^66^ Surface modifications to titanium implants are still necessary to promote osteogenesis, osteoconduction, and osseointegration. Electrospinning has been used as a simple method to produce nanofibrous structural materials in recent years. In orthopedic implants, electrospun piezoelectric membranes have also been utilized as grafts to replace injured cartilage.^67^ Higher rates of cell adhesion and proliferation were seen on the dental implant surface covered with PLGA/collagen fibers, which also promoted more mineralization, differentiation, and proliferation of cells. More surface area for fibroblast adhesion is another advantage of electrospun nanofiber coatings.^68^ Although biofilm can result in periimplantitis and dental implant loss, these implants have become popular choices for dental prosthesis. Researchers have been looking into ways to make implants with an osteointegrative surface while minimizing the establishment and production of biofilms.^69^ PCL/tetracycline nanofibers (5, 10%, and 25% wt) were tested for their antibacterial activity against periimplantitis-associated microorganisms, such as *P. intermedi*a, *P. gingivalis, A. actinomycetemcomitans* and* F. nucleatum,*. The biofilm of these bacteria was completely inhibited by nanofibers containing 25% wt tetracycline.^70^

## Wound Healing

 Historically, treatments for bone lesions and wounds have involved autogenous, allogenic, and xenogenous materials. There is a pressing demand for more reliable and effective materials, particularly for chronic wounds.^71^ Regenerative medicine has emerged as a promising alternative, being a multidisciplinary field focused on restoring, treating, and regenerating tissues and organ functions. It achieves this by creating a controlled environment that encourages and guides cell proliferation and new tissue formation.^72^

 Numerous studies have been conducted on electrospun fiber mats as wound dressings that can provide regenerative and antibacterial properties. The use of such wound dressings as delivery vehicles for analgesics and antibiotics, such as topical anaesthesia, might reduce the amount of systemic administration required and, as a result, reduce their numerous undesired side effects.^73^ Similar to how topical anaesthesia and antibiotics may be applied to surgical or traumatic wounds in dentistry, electrospun mats can be utilised. Electrospun fibres might be used as dressings for surgical wounds or oral mucosal lesions, such as ulcers, in addition to wound healing.^74^ It has been noted that polymers such as chitin and PLLA serve as efficient scaffolds for the proliferation and differentiation of human mucosal cells. When evaluated *in vitro* against rat mucosal cells, electrospun silk fibroin has more recently demonstrated potential comparable to human skin matrices.^75^ A nanofiber made from electrospun PCL and gum tragacanth loaded with curcumin was shown to increase curcumin’s bioavailability, accelerate wound healing, and promote fibroblast growth and collagen formation.^76^ Fibrous scaffolds and dressings for oral mucosal abrasions have a promising future, although further research is undoubtedly needed to determine their effectiveness. These substances also exhibit great promise in the treatment of other mucosal disorders.^77^

## Drug Delivery

 Like all surgical specialties, dentistry requires the use of medications such as analgesics and antibiotics both before to and following surgery. As previously mentioned, drug delivery devices that employ electrospun scaffolds can reduce systemic dose. Electrospun scaffolds have been used to deliver anti-inflammatory treatments, antibacterial medications, and analgesics.^78^ Growth factor-releasing scaffolds and implantable drugs that promote wound healing by speeding osseointegration and/or reducing infection risk are examples of more recent uses for electrospun scaffolds.^79^ In addition to having superior mechanical qualities over unmodified fibers, PLLA fibers with growth factor-loaded nanodiamonds also transport and administer growth factors and medications that promote bone mending, lower inflammation, and prevent infection. These scaffolds may also be utilized to monitor and assess guided tissue regeneration at the cellular level using a variety of imaging methods since the nanodiamonds can be made luminous.^80^ Table 2 represents some drugs and polymers with their potential applications.

**Table 2:** Overview of different nanofibers developed by interactions of polymer and drug with their applications

**S. No.**	**Polymers**	**Drug**	**Application**	**Inference**	**Ref**
1.	PLA, PEVA and PLA/PEVA	Tetracycline hydrochloride	Treatment of periodontal disease	poly(ethylene-co-vinyl acetate) (PEVA), Poly(lactic acid) (PLA), or a 50:50 blend of the two were used to create the mats using a single nozzle technique. To solubilize the medication, the fibers were electrospun from chloroform solutions that contained a small quantity of methanol.	^81^
2.	Poly(L-lactide-co-D,L-lactide) (PLA) and polycaprolactone	Metronidazole	Periodontitis treatment	In the treatment of localized periodontitis, coaxial electrospun nanofibers showed enhanced sustained metronidazole release and may be employed as efficient drug delivery systems.	^78^
3.	Cellulose acetate	Chlorhexidine	Treatment of dental diabetic wounds	The encapsulation and controlled release of chlorhexidine were accomplished effectively. *E. faecalis* and *S. mutans* were successfully eradicated by the mats.	^82^
4.	Poly(DL-lactide-ε-caprolactone) Gelatin	Tetracycline hydrochloride	Anti-bacterial dental implant coating.	Fibers containing tetracycline have a lot of potential as an antibacterial coating for dental implants since they successfully stop the growth of microorganisms linked to peri-implantitis.	^83^
5.	Poly(methylmethacrylate)/Poly (ethyleneoxide)	Doxycycline	Antibacterial activity against an oral pathogen	Poly (ethyleneoxide) produced effective drug release systems with antibacterial activity against *S. mutans* when it was introduced to PMMA fiber mats at 20% and 30%.	^84^
6.	polycarbonate urethane (PCNU)	Ciprofloxacin	Gingival tissue engineering when a local antibiotic dosage is required	Electrospun polymer scaffoldsthat provide sustained release of Ciprofloxacin over time and helps ingingival tissue engineering.	^85^
7.	Poly(caprolactone)	Oxytetracycline Zinc oxide nanoparticles	treatment of periodontal disease	Oxytetracycline and Zinc oxide nanoparticles were successfully incorporated into Poly(caprolactone) nanofibers using the electrospinning technique, which produced an Oxytetracycline release that lasted for up to five days.	^86^
8.	Poly(caprolactone)	Metronidazole nanoparticlesand hidroxyapatite	anti-infective guided bone regeneration membranes	For anti-infective directed bone regeneration membranes, electrospun core-shell nanofibers were developed that have two properties: they inhibit the release of Metronidazole and promote osteogenesis.	^87^
9.	Poly(caprolactone)/Gelatin	DoxycyclineHyclate Hydroxyapatite Nanoparticles	anti-tumoral and antibacterial activity	Electrospun poly-ε-caprolactone-gelatin composite nanofibers were employed as a co-delivery vehicle for hydroxyapatite and doxycycline nanoparticles. According to our research, the co-delivery method of hydroxyapatite and doxycycline nanoparticles has two therapeutic benefits.	^88^
10.	Poly(DL-lactide)/ Poly(ε-caprolactone)/Gelatin/ HexamethyldisilaneTitanium	Tetracycline	antimicrobial surface modifier and osteogenic inducer for Ti dental implants.	Tetracycline-incorporated polymer nanofibers may be used as an antibacterial surface modification and osteogenic inducer for titanium dental implants.	^89^
11.	Polyvinylpyrrolidone/Poly(methyl methacrylate)	Cetylpyridinium chloride Miconazole	antifungal action against Candida albicans	Because of their regulated release and long-lasting effects, Cetylpyridinium chloridecontaining nanofibers are useful in preventing the growth of Candida albicans and may be utilized as an alternate antifungal treatment, possibly increasing patient adherence.	^90^

## Patents

 For creating a nano-crystalline apatite, Dziuron et al.^91^ were granted a patent. The compositions including nano-crystalline apatite that are suitable as bone-restorative materials, or ideally as tooth-restorative materials, are described in this invention. The material produced can be used as bone cements or bone substitutes, and especially as fillings, inlays or on lays, luting cements, veneers for crowns and bridges, materials for artificial tooth, dentine- and enamel bonding agents, underfilling materials, core build-up materials, sealants, root canal filling materials or other miscellaneous curable materials in prosthetic, conservative and preventive dentistry. A patent was granted to Yong et al.^92^ for the development of a dental membrane. According to their invention, a dental membrane is realized by using a nanofiber web made of a biodegradable polymer, and then is decomposed a predetermined time, and thus there is an advantage of eliminating the need for a separate operation for removing the membrane.

 The process for producing dental floss employing a nanofiber composite filament with a large specific surface area and three-dimensional pores that can successfully impregnate a medicament, such as a hemostatic agent, with the nanofiber composite filament is described in patent application CN111012958A. Finally, when such a dental wire is applied, bleeding can be effectively stopped and the gum can be contracted (converged) when the gum is opened.^93^

 Kim et al.^94^ were granted a patent for their technique of producing a dental cord using the nanofiber multiple yarn according to the invention has a relatively small diameter of the fiber compared to that of a conventional material, and thus has a large specific surface area and a large number of three-dimensional pores, to accordingly effectively impregnate a drug such as a hemostatic agent by using the nanofiber multiple yarn. As a result, applying such a dental cord effectively achieves a hemostatic and gingival contraction (convergence) effect when the gingiva is opened.

 A patent was granted to Jian et al.^95^ for creating calcium-phosphate nanofiber matrices made of crystalline calcium-phosphate nanofibers scattered at random. Sol-gel techniques in conjunction with electrospinning are used to create the nanofibers. The nanofibers might be solid, hollow, or made up of a calcium-phosphate shell enclosing an inner core of a polymer to which biologically useful additives could be added. In addition to being used as implants to treat bone, dental, or periodontal illnesses and abnormalities, the nanofiber matrices can be employed to cultivate bone and dental cells.Application for a patent KR102460064B1 offers a dental mask that overcomes the disadvantages of the existing disposable masks with traditional Korean paper and nanofibers to provide a skin-friendly inner skin layer, a hydrophilic intermediate filter layer using nanofibers and impregnated nanoparticles, and self-cleaning by visible light (It relates to a method of manufacturing a recycled nanofiber dental mask composed of a triple filter with a hydrophobic outer filter layer that can self-cleaning and can simultaneously diagnose environmental pollution (air/bacteria, virus) and sterilize by visible light at the same time.^96^ Won-youl et al.^97^ received recognition for creating a nanofiber chip with an average diameter of 10 to 1000 nm. In particular, in the dental application field, the drug has been loaded using polymer material with pores to suppress inflammatory expression of periodontal pocket after treatment of periodontal disease, but its structural characteristics are unclear, and the amount of released drug is continuously released. The problem to be solved by this invention is that nanofiber is made of a biodegradable polymer material or titanium oxide, the drug is mounted on the surface and pores of the nanofiber can be used for disinfection, antibiotics, etc., the diameter of the fiber Due to its nano size, it has a very high specific surface area and a network (network) structure makes it easy not only to adhere to the wound site but also to release the contained drug for a certain period of time. And to provide a method for producing the same. According to patent application CN113768796A, a fiber reinforced light curing repair material, and particularly provides a resin-based repair material reinforced by nano fibers and a preparation method thereof. The nano-fiber is prepared by a melt spinning technology. When the resin-based repair material is applied to dental repair, the curing method is photocuring.^98^ The summarized patented nanofibers in dental regeneration are listed in Table 3.

**Table 3:** Patented Nanofibers in Dentistry

**PATENT NO.**	**TITLE**	**INFERENCE**	**APPLICATION **	**Ref**
EP1746966B1	Composition containing nano-crystalline apatite	Crystalline calcium-phosphate nanofibers contain nano-crystalline apatite and are useful as bone- or, preferably, tooth restorative materials.	Bioactive fillers in restorative materials Endodontic sealing and remineralizing activity Periodontal bone regeneration	^91^
US2018078346A1	Dental membrane	A dental membrane capable of controlling the decomposition rate of a laminated support, thereby maximizing the function of the dental membrane and improving skin adhesion.	Periodontal GTR Alveolar GBR Ridge preservation	^92^
CN111012958A	Method for manufacturing dental thread using nanofiber composite yarn	It is characterized by comprising a drug-impregnated nanofiber composite yarn obtained by doubling and twisting at least 2 nanofiber tape yarns	Drug-loaded floss for localized therapy Periodontal pocket drug delivery	^93^
US2017312056A1	Dental cord using nanofiber conjugate yarn, and manufacturing method therefor	A dental cord using a nanofiber multiple yarn having a large specific surface area and a large number of three-dimensional pores, thereby effectively impregnating a drug such as a hemostatic agent, and a method of manufacturing the dental cord.	Gingival retraction Hemostatic control Drug delivery (anti-inflammatory/antimicrobial) Implant soft tissue retraction	^94^
US2009317446A1	Calcium Phosphate Nanofibers	Crystalline calcium-phosphate nanofibers are arranged in random patterns to form calcium-phosphate nanofiber matrices.	Bone graft scaffolds Periodontal tissue regeneration Implant osseointegration improvement	^95^
KR102460064B1	A nano fiber dental mask and a method of manufacturing the same	A traditional Korean paper that is safe for the skin and free of chemicals, as well as a dental mask with a layer of nanofiber laminated on it	Enhanced Filtration for Dental Procedures Improved Breathability and Comfort Reusable / Sterilizable Mask Designs	^96^
WO2014148765A1	Dental nanofiber chip equipped with medication and method for manufacturing same	A nanofiber chip was manufactured having a very large specific surface area, and the network structure thereof enables easy attachment to a wounded area. The contained medication can be continuously released for a predetermined period.	Endodontic antimicrobial release Peri-implantitis treatment Regenerative dental therapy Caries control and remineralization	^97^
CN113768796A	Nanofiber reinforced resin-based repair material and preparation method thereof	Provides a fiber reinforced light curing repair material, and particularly provides a resin-based repair material reinforced by nano fibers, and can be used for preparing restorations such as crowns, bridges, inlays, and the like in clinical applications.	Direct filling restorations Tooth fracture restoration Minimally invasive restorations Crown/bridge repair	^98^

## Clinical Study

 A low-dose controlled-release delivery method was developed by Chaturvedi TP et al.^99^ to treat periodontal infections. Doxycycline (DOX)-containing Poly e-caprolactone (PCL) nanofibers were effectively electrospun to create a novel sustained release medication system that was clinically assessed for periodontal disorders. Two experimental treatment groups, Group A-SRP (Scaling and Root Planing) + DOX nanofibers and Group B-SRP (Scaling and Root Planing) alone (control group), were assigned forty sites from seven patients (four females and three males) with chronic periodontitis (5-8mm probing depth). The gingival index (GI), plaque index (PI), and probing depth (PD) of each of these individuals were clinically assessed. The drug-coated nanofibers followed first order release and had a sustained impact for up to 11 days (264 hours). In comparison to the control group, the combination of SRP (Scaling and Root Planing) + DOX nanofibers (Group A) produced additional advantages^.^

 To treat periodontal infections, Chaturvedi TP et al.^100^ attempted to develop a low-dose controlled-release delivery method. Poly e-caprolactone (PCL) nanofibers containing metronidazole (MET) were effectively electrospun to produce a novel sustained release medication system that was clinically assessed for periodontal disorders. It was demonstrated that the retentive nanofibers provide a regulated medication delivery system. MET was used in PCL to design nanofibers using the electrospinning method. Seven patients with chronic periodontitis (three men and four women) had forty sites (5–8mm probing depth) divided into two experimental therapy groups: Group A received SRP + MET nanofibers, while Group B received SRP alone (control group). The gingival index (GI), plaque index (PI), and probing depth (PD) of each of these individuals were clinically assessed. The drug-coated nanofibers had a first-order release and maintained their effects for 11 days (264 hours).

 The therapeutic efficacy of *Occimum sanctum *in the treatment of periodontal disease is the subject of limited study. To avoid systemic circulation and achieve the highest concentration at the location that needs the treatment, George et al.^101^ designed local drug delivery methods that allowed for direct delivery to the sick site. The study’s objective was to examine how *O. sanctum *affected the anti-inflammatory efficacy of interleukin 1-β (IL-1β) in patients suffering from periodontitis. To compare the clinical parameters and IL-1β levels of subjects who received nonsurgical periodontal therapy in addition to local application of *O. sanctum* fibers versus those who received nonsurgical periodontal therapy alone, a randomized, controlled clinical trial was carried out with a sample size of 15 patients. At one month, neither the IL-1β levels nor the clinical parameters between the test and control groups showed a statistically significant difference, according to the current study. Clinical attachment-level gain varied significantly between groups (*P* = 0.046). The effects of *Occimum sanctum* nanofibers showed a decrease in IL-1β levels. This study indicates that using O. sanctum as an adjuvant to treat periodontitis has additional benefits. Different clinical trials are summarized in the Table 4.

**Table 4:** Study design, Sample Size, Conditions, and Outcome Observed of different clinical trials on application of nanofibers in dentistry.

**TRIAL NO. **	**TITLE **	**STUDY DESIGN**	**SAMPLE SIZE **	**CONDITIONS **	**OUTCOME OBSERVED**	**REF**
NCT07149493	Probiotic Nanofiber Floss and Subgingival Pathogen Suppression	Randomized	33	Dental Hygiene Probiotics, Periodontitis Prevention of Dental Caries Gingivitis and Periodontal Diseases Periodontal Health Oral Microbiome	Change in Approximal Plaque Index and Change in Sulcus Bleeding Index was observed.	^102^
NCT03690960	Antimicrobial Effect of Modified Antibiotic Nanofibers for Regenerative Endodontics Procedures	Randomized	30	Necrosis, pulp	Antimicrobial effect was studied	^103^
CTRI/2024/04/065742	Evaluation of nanofiber scaffolds laden *Ashvakatri* in the management of chronic periodontitis-a randomized, controlled split pocket study	Randomized	31	Chronic periodontitis	Plaque index, Gingival index, Probing pocket depth, and Clinical attachment level	^104^
IR.IUMS.REC.1401.355	Self-assembling peptide nanofibers and nanoceramics in a model of alveolar bone repair: Insights from in vivo experiments and clinical trial	Randomized	9	Alveolar bone repair	Soft tissue healing, and CBCT analysis	^105^

## Conclusion

 Electrospun nanofibers are among the most versatile and biomimetic materials in modern dental regenerative medicine. They provide structural, mechanical, and biological benefits that closely mimic the natural extracellular matrix. Their high surface area, adjustable porosity, and capacity to carry bioactive molecules, enable them to support essential regenerative processes, including cell adhesion, proliferation, differentiation, angiogenesis, and mineralization. In various dental applications, including pulp-dentin regeneration, periodontal treatment, alveolar bone repair, implant surface coating, oral wound healing, and localized drug delivery, electrospun nanofibers outperform traditional materials. Their ability to simultaneously deliver antibiotics, growth factors, nanoparticles, and stem cell-stimulating agents within a single matrix enhances their therapeutic potential. Overall, these developments position electrospun nanofibers as a groundbreaking platform for improving the precision, efficacy, and biological integration of dental therapies.

## Future Perspective

 Despite notable advances, various challenges still hinder their broad clinical adoption. Key issues include enhancing mechanical stability in the mouth’s moist environment, ensuring consistent fabrication at scale, and ensuring long-term biocompatibility alongside safe degradation. Dental tissues pose additional regenerative challenges due to their complex hierarchical structure and interactions with microbes, requiring more advanced scaffold designs. Future efforts should focus on developing smart nanofibers that can respond to physiological signals, such as pH changes, enzymes, bacteria, or mechanical forces. Promising innovations encompass multilayered and 3D electrospun structures, hybrid scaffolds that incorporate ceramics or peptides mimicking growth factors, and fibers that modulate immune responses to guide tissue regeneration. Moreover, integrating electrospinning with digital dentistry, 3D printing, and stem-cell techniques could facilitate the creation of patient-specific scaffolds tailored to individual needs and regenerative objectives.

 As collaboration across biomaterials science, dentistry, tissue engineering, and clinical research expands, electrospun nanofiber systems are set to revolutionize regenerative strategies in dentistry. Their versatility, biocompatibility, and therapeutic multifunctionality position them as a key technology for future dental regeneration, promising to translate laboratory innovations into safe, effective solutions that deliver minimally invasive, impactful clinical outcomes.

## Competing Interests

 The authors report no financial or any other conflict of interest in this work.

## Ethical Approval

 Not applicable.
